# Circulating SCGB3A2 Is Associated With Impaired Coronary Collateralization in Patients With Chronic Total Occlusion and Type 2 Diabetes: A Proteomic Discovery and Validation Study

**DOI:** 10.1111/1753-0407.70281

**Published:** 2026-10-15

**Authors:** Jian‐Jun Chen, Shuai Chen, Jia‐Kang Wang, Ke Huang, An Wang, Lin Lu, Yang Dai, Feng‐Hua Ding

**Affiliations:** ^1^ Department of Cardiovascular Medicine Rui Jin Hospital, Shanghai Jiao Tong University School of Medicine Shanghai People's Republic of China; ^2^ Institute of Cardiovascular Diseases, Shanghai Jiao Tong University School of Medicine Shanghai People's Republic of China

**Keywords:** chronic total occlusion, coronary collateralization, Olink proteomics, risk stratification, secretoglobin 3A2, type 2 diabetes mellitus

## Abstract

**Background:**

Type 2 diabetes mellitus (T2DM) is associated with impaired coronary collateralization in patients with chronic total occlusion (CTO), but circulating biomarkers reflecting defective collateral remodeling remain poorly defined.

**Methods:**

Olink proteomic profiling was performed in a discovery cohort of 88 CTO patients with T2DM stratified by collateral status. Candidate proteins were prioritized using nominal differential expression and partial least squares‐discriminant analyses. Secretoglobin family 3A member 2 (SCGB3A2) was selected for validation by ELISA in an independent cohort of 832 CTO patients (425 with T2DM and 407 without diabetes). Associations with collateralization and incremental predictive value beyond clinical risk factors were evaluated.

**Results:**

Among 265 proteins, eight showed nominal differential expression, with SCGB3A2 exhibiting the strongest signal and highest variable importance. In the T2DM cohort, serum SCGB3A2 levels were significantly higher in patients with poor collateralization and inversely correlated with Rentrop grades (both *p* < 0.001). SCGB3A2 remained independently associated with poor collateralization after multivariable adjustment (OR per 10 ng/mL increase, 1.63; 95% CI, 1.39–1.94; *p* < 0.001). Addition of SCGB3A2 to a clinical model improved discrimination with an increase in C‐statistic from 0.73 to 0.78 and reclassification (NRI 0.62; IDI 0.08). Similar associations were observed in patients without diabetes.

**Conclusions:**

Circulating SCGB3A2 is independently associated with impaired coronary collateralization in CTO patients. It may reflect biological processes underlying collateral failure and improve risk stratification, particularly in patients with T2DM.

## Highlight

- An integrated proteomics‐to‐validation strategy identified SCGB3A2 as a novel biomarker independently associated with impaired coronary collateralization in patients with chronic total occlusion and Type 2 diabetes.
- These findings provide new insights into the association between SCGB3A2 and impaired coronary collateralization in diabetes and support its potential for improving risk stratification in patients with chronic total occlusion.

## Graphical abstract

Proteomic profiling identified SCGB3A2 as a novel biomarker associated with impaired coronary collateralization in patients with chronic total occlusion and Type 2 diabetes. Independent validation supports its potential for risk stratification.

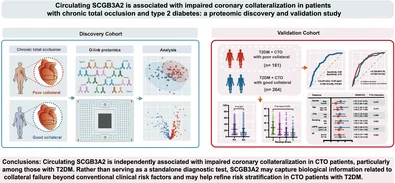

## 1 Introduction

Chronic total occlusion (CTO) represents a severe form of coronary artery disease characterized by complete and persistent obstruction of a major epicardial coronary artery, resulting in chronic myocardial ischemia [1]. Coronary collateral circulation is an important adaptive response in this setting, as collateral vessels may restore distal perfusion, limit ischemic injury, preserve ventricular function, and improve clinical outcomes in patients with CTO [2, 3, 4]. Conversely, insufficient collateral development is associated with greater ischemic burden and adverse cardiovascular outcomes [5, 6, 7, 8].

Type 2 diabetes mellitus (T2DM) is one of the clinical conditions most strongly associated with impaired coronary collateralization in CTO patients [6]. The development of functional collateral vessels is not merely a process of capillary sprouting, but a coordinated adaptive vascular remodeling response involving endothelial activation, inflammatory cell recruitment, shear stress–mediated arteriogenesis, angiogenic signaling, and extracellular matrix remodeling. In T2DM, hyperglycemia, oxidative stress, chronic low‐grade inflammation, endothelial dysfunction, and impaired reparative cell function may collectively disrupt this response, resulting in inadequate collateral maturation despite persistent ischemic stimulation. Although therapeutic strategies aimed at enhancing angiogenesis and arteriogenesis have been extensively investigated, their clinical efficacy remains limited [9, 10, 11]. These observations highlight the need to better understand the molecular regulators of ischemia‐induced collateral remodeling and collateral failure in patients with T2DM and CTO [12].

Circulating biomarkers may provide clinically useful information on the biological processes underlying impaired collateral development and may help refine risk stratification in this high‐risk population. However, molecular markers that reflect defective collateral remodeling in CTO patients with T2DM remain incompletely defined. High‐throughput proteomic profiling offers an unbiased approach to identify circulating proteins associated with vascular dysfunction, inflammation, and maladaptive repair responses [13]. In the present study, we used Olink proximity extension assay–based proteomics to screen for candidate proteins associated with poor collateralization in CTO patients with T2DM, followed by validation using enzyme‐linked immunosorbent assay (ELISA) in an independent cohort. We aimed to identify novel circulating biomarkers associated with impaired coronary collateralization and to evaluate their potential incremental value for risk stratification in CTO patients, with a particular focus on those with T2DM, while also exploring whether the association extends to nondiabetic patients.

## 2 Methods

### 2.1 Study Design

This is an observational biomarker discovery and validation study, comprising an initial biomarker discovery phase where serum biomarkers that best distinguish patients with poor collateralization (PC) from those with good collateralization (GC) were identified. The best discriminating biomarkers from the discovery cohort were validated using ELISA in a subsequently recruited prospective validation cohort with CTO. The validation cohort was stratified according to diabetes status, including 425 CTO patients with T2DM, which served as the primary validation cohort, and 407 CTO patients without T2DM, which were analyzed as an additional comparison cohort.

### 2.2 Study Population

The discovery cohort included consecutively enrolled patients diagnosed with either poor or good collateralization, all of whom were newly diagnosed CTO cases visiting the catheterization laboratory of cardiology at Ruijin Hospital between May 2018 and January 2019. The validation cohort initially consisted of 978 consecutive patients with stable angina pectoris and at least one chronic coronary occlusion (> 3 months) in a major epicardial artery, screened from the Ruijin Hospital (Shanghai) percutaneous coronary intervention outcomes database between March 2019 and April 2025. After applying exclusion criteria, 146 patients were excluded due to percutaneous coronary intervention (PCI) within the preceding 3 months (*n* = 50), history of coronary artery bypass graft surgery (*n* = 42), pulmonary heart disease (*n* = 16), chronic heart failure (*n* = 19), immune system disorders or malignant tumor (*n* = 11), and renal impairment requiring dialysis (*n* = 8). No patients were excluded because of missing angiographic or SCGB3A2 data. Thus, the final validation cohort comprised 832 patients (425 diabetic and 407 nondiabetic). The T2DM cohort was used as the primary validation dataset to maintain consistency with the discovery cohort. Type 2 diabetes mellitus was diagnosed according to the American Diabetes Association criteria [14]. The diagnosis of stable angina pectoris adhered to the criteria recommended by the American College of Cardiology/American Heart Association [15]. The duration of coronary occlusion was estimated based on information obtained from previous coronary angiography, history of myocardial infarction in the territory supplied by the occluded vessel, or the first onset of existing angina that suddenly worsened. All patients in this study were enrolled from the clinical trial registered at ClinicalTrial.gov (NCT06137521), and written informed consent was obtained from all patients.

### 2.3 Acquisition of Olink Proteomics Data

Preprocedural plasma samples from 88 CTO patients (baseline demographic and clinical characteristics can be seen in Table S1) in the discovery cohort of this study were used for Olink proteomics analysis. Protein biomarkers were measured using multiplex proximity extension assays (PEA) with the Olink Target Cardiovascular II, Cardiovascular III, and Cardiometabolic panels (each panel contains 92 proteins, totaling 276 proteins). Experiments strictly followed the manufacturer's standardized operating procedures. Briefly, samples were incubated with a pair of antibodies (PEA probes) carrying unique DNA barcodes. The presence of the target protein brings the two probes into proximity, allowing a proximity extension reaction to form a specific DNA template. This DNA template was then amplified and quantified using a microfluidic real‐time PCR system. For data quality control and preprocessing, samples with an internal control coefficient > 0.5 were excluded to ensure data reliability. All protein detection values were converted to normalized protein expression (NPX) values. NPX is a relative quantification unit on a Log2 scale, enabling comparison across samples.

For quality control (QC) of each plate, four internal control samples are first added to every plate. The standard deviation (SD) of these internal control samples within each plate is then assessed. Only plates with an internal control SD below 0.2 NPX are reported. Subsequently, the median deviation of each individual sample relative to the control sample median is evaluated. Samples exhibiting a median deviation less than 0.3 NPX pass QC. Following QC procedures, 11 proteins failing QC were excluded, resulting in the inclusion of 265 proteins for analysis.

### 2.4 Serum Samples

Fasting venous blood samples were collected from all participants on the morning of coronary angiography after ≥ 8 h overnight fasting. Serum specimens were immediately aliquoted and cryopreserved at −80°C pending biochemical analysis. Serum concentrations of glucose, creatinine, triglycerides, total cholesterol, low‐density lipoprotein (LDL)‐cholesterol, and high‐density lipoprotein (HDL)‐cholesterol were quantified using standardized automated assays (Hitachi 912 analyzer, Roche Diagnostics GmbH, Mannheim, Germany). Estimated glomerular filtration rate (eGFR) was derived via the CKD‐EPI equation:

eGFR (mL/min/1.73 m^2^) = 141 × min(creatinine/*κ*, 1)*α* × max(creatinine/*κ*, 1)−1.209 × 0.993Age × [1.018 if female] [*κ* = 0.7 (females)/0.9 (males); *α* = −0.329 (females)/−0.411 (males); min = minimum of SCr/*κ* or 1; max = maximum of SCr/*κ* or 1] [16]. Serum creatinine values measured in μmol/L were converted to mg/dL prior to calculation using the conversion factor of 88.4 μmol/L = 1 mg/dL.

Serum SCGB3A2 levels were measured using a commercially available ELISA kit according to the manufacturer's instructions (CSB‐EL020819HU; CUSABIO) and performed blinded to collateral status and clinical characteristics. The recommended detection range of the kit was 46.88 pg/mL–3000 pg/mL, and the sensitivity was 11.72 pg/mL. Regarding kit precision, the average intra‐assay coefficient of variation and inter‐assay coefficient of variation were < 8% and 10%, respectively. Prior to assay, serum samples were diluted 1:100 with dilution buffer. The optical density of each sample was determined using a microplate reader at 450 nm. SCGB3A2 levels were calculated using the optical density values of the standard curve and reported concentrations were back‐calculated by multiplying by the dilution factor.

Variables with missing values < 5% were analyzed using complete‐case analysis. No variable exceeded 5% missingness.

### 2.5 Coronary Angiography

Coronary angiography was conducted through femoral or radial arterial access utilizing 6F diagnostic catheters. Quantitative coronary analysis was independently performed by two blinded experienced cardiologists, who were unaware of both the study protocol and biochemical assay results. The development of collateral circulation perfusing the distal territory of totally occluded coronary arteries was visually assessed using the Rentrop classification [17] system: Grade 0 (no detectable collaterals), Grade 1 (faintly opacified collaterals with poorly visualized distal vessels), Grade 2 (partial collateral vessel filling), and Grade 3 (complete collateral filling). Subjects were subsequently stratified into poor collateralization (PC; Rentrop grades 0–1) and good collateralization (GC; Rentrop grades 2–3) groups. The resulting group sizes reflected the observed distribution of collateralization in the study population rather than preferential sampling. Any interpretive discrepancies underwent adjudication by a third reviewer.

### 2.6 Biomarker Discovery

For biomarker discovery in the proteomics data, we employed a dual analytical approach. Univariate analysis assessed differential expression of each protein between PC and GC groups using Student's *t*‐test. Because the discovery phase was intended to generate candidate biomarkers for subsequent validation rather than establish definitive proteomic associations, proteins with nominal *p* < 0.05 were considered exploratory candidates for downstream validation despite the absence of statistical significance after multiple‐testing correction. Results were visualized through volcano plots depicting log2 fold‐change (FC) for magnitude of difference and ‐log10(*p*‐value) for statistical significance, with UniProt IDs mapped to gene symbols using the org.Hs.eg.db database in R software (version 4.5.1). Multivariate analysis involved performing partial least squares discriminant analysis (PLS‐DA) to identify global protein expression patterns discriminating PC and GC groups. Key steps included model optimization, where the optimal number of components (max = 5) was determined by 10‐fold cross‐validation with 50 repeats to minimize the balanced error rate (BER); visualization of sample distribution in the latent variable space (Components 1–2) with 95% confidence ellipses [18]. A multivariable logistic regression analysis was performed using SPSS 27.0 for macOS (SPSS Inc., Chicago, IL, USA) to evaluate the association between the candidate proteins and collateral circulation. Key packages included mixOmics (v6.26.0) for PLS‐DA, AnnotationDbi and org.Hs.eg.db for annotation, and ggplot2 (v4.0.1) for visualization.

### 2.7 Statistical Analysis

Continuous variables were presented as mean ± SD for normally distributed data and as median (25th–75th percentile) for nonnormally distributed data, whereas categorical variables were expressed as frequencies (percentages). Differences between two groups were assessed using the Student's *t*‐test for normally distributed continuous variables, the Mann–Whitney U test for nonnormally distributed continuous variables, and the *χ* ^2^ test for categorical variables. For validation‐phase biomarker assessment, 2 multivariate logistic regression models were constructed to assess the independent determinants of PC: the traditional risk factors for poor collateralization were included in Model 1, and serum SCGB3A2 levels (per 10 ng/mL) were additionally included in Model 2. The traditional risk factors in both models included age, gender, body mass index (BMI), hypertension, smoking, eGFR, total‐to‐HDL cholesterol ratio, glycated hemoglobin A1c (HbA1c), log10‐transformed high sensitivity C‐reactive protein (hs‐CRP), left ventricular ejection fraction (EF). Calibration performance and internal validation were assessed using bootstrap resampling with 1000 iterations with the rms package (V8.1‐1). Decision curve analysis (DCA) was conducted using the rmda package (V1.6). Receiver operating characteristic (ROC) curve analyses were performed using serum SCGB3A2 levels and the predicted probabilities (C‐statistic) from the regression models (with/without serum SCGB3A2 levels [per 10 ng/mL]), comparing the areas under the curve (AUC) using the pROC package (v1.19); category‐free net reclassification improvement (NRI) was assessed with the nricens package (v1.6); integrated discrimination improvement (IDI) was calculated with the Hmisc package (v5.2.5) in R software. The optimal cutoff value of circulating SCGB3A2 was determined using the maximum Youden index (sensitivity + specificity − 1) from ROC curve analysis, separately for patients with and without T2DM. Differences among groups were initially evaluated by one‐way analysis of variance (one‐way ANOVA). Given a significant overall group effect, post hoc multiple pairwise comparisons were performed with Bonferroni correction to control Type I error. All analyses used two‐tailed tests with an overall significance level (*α*) of 0.05.

## 3 Results

### 3.1 Processing and Presentation of Proteomics Data

We designed a flowchart (Figure 1A) to screen for biomarkers that could diagnose the extent of collateral circulation development in CTO patients with T2DM. We performed Olink proteomics analysis on a cohort of patients (*n* = 88) clinically diagnosed with CTO coronary artery disease and T2DM between 2018 and 2019. Cases in this cohort were classified based on Rentrop score: patients with scores 0–1 and 2–3 were categorized as poor collateralization (PC, *n* = 41) and good collateralization (GC, *n* = 47) groups, respectively.

**FIGURE 1:**
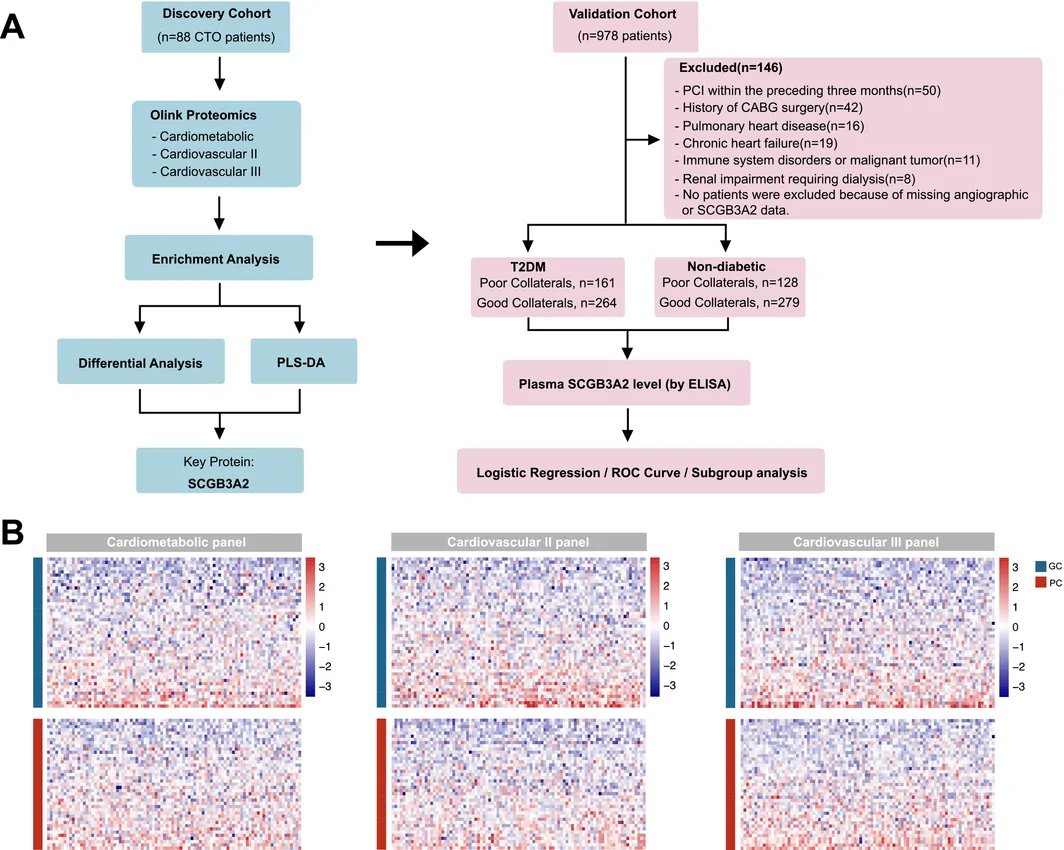
Discovery and validation workflow of collateral circulation biomarkers in CTO patients. (A) Flowchart of the biomarker discovery and validation process. Coronary collateral circulation was assessed in patients with chronic total occlusion (CTO). Potential biomarkers were identified through proteomic profiling (Olink), followed by differential expression analysis and partial least squares‐discriminant analysis (PLS‐DA). Candidate proteins were then validated by enzyme‐linked immunosorbent assay (ELISA) in an independent cohort. (B) Heatmaps showing *Z*‐score normalized proteomic data, stratified by collateral circulation grade. Samples are arranged in descending order of total *Z*‐score within each group. CTO, chronic total occlusion; CABG, coronary artery bypass graft; ELISA; enzyme‐linked immunosorbent assay; PCI, percutaneous coronary intervention; PLS‐DA, partial least squares discriminant analysis; ROC, Receiver operating characteristic; SCGB3A2, secretoglobin family 3A member 2.

We measured 265 protein biomarkers using multiplex PEA with Olink Target Cardiovascular II, III, and Cardiometabolic panels, and expressed the data as NPX. After *Z*‐score normalization, samples were grouped into PC and GC categories, and heatmaps were generated to visualize protein expression (Figure 1B). The results showed distinct differences in protein expression between the PC and GC groups, indicating potential biomarkers for predicting collateral circulation development.

### 3.2 Proteomic Screening for Candidate Biomarkers

To identify potential biomarkers, we merged the NPX values from three panels and categorized proteins into two groups (PC and GC). Exploratory proteomic analysis identified 8 proteins showing nominally significant differences between the PC and GC groups (all nominal *p* < 0.05), including SCGB3A2, which had the smallest nominal P and the most pronounced differential expression (Figure 2A,B, Table S2). However, these associations did not remain statistically significant after false discovery rate correction, likely due to the relatively limited sample size of the discovery cohort. We further performed partial least squares discriminant analysis (PLS‐DA), which demonstrated separation between the PC and GC groups based on global proteomic profiles (Figure 2C). Permutation testing with 1000 random permutations showed that the original model significantly outperformed permuted models (BER = 0.376, permutation *p* = 0.024), indicating that the observed separation was unlikely to occur by chance (Figure S1B). Ten proteins with VIP scores > 1.5 were identified, among which SCGB3A2 showed the highest VIP score (Figure 2D). A Venn diagram confirmed that the differentially expressed proteins were included in the VIP set (Figure 2E), suggesting their biological relevance. Violin plot analysis further confirmed significantly higher SCGB3A2 levels in the PC group than in the GC group (Figure 2F).

**FIGURE 2:**
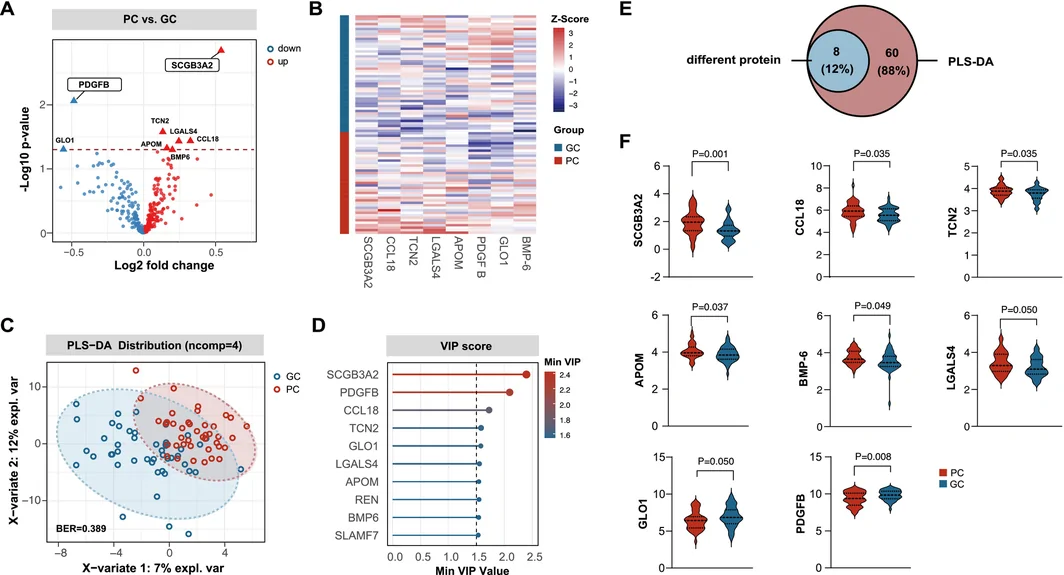
Proteomic profiling and biomarker identification for collateral circulation in CTO. (A) Volcano plot of differential protein expression between poor collateralization (PC) and good collateralization (GC) groups. Two‐tailed *t*‐tests were used. (B) Heatmap of the eight differentially expressed proteins, with samples ordered as in panel A. (C) Partial least squares‐discriminant analysis (PLS‐DA) score plot based on 265 unique proteins, showing separation between PC and GC groups along the first two components. Ellipses indicate 95% confidence intervals. Included proteins in the first two components can be viewed in Figure S1A. (D) Variable importance in projection (VIP) scores from the PLS‐DA model. Proteins with VIP > 1.5 are highlighted. (E) Venn diagram illustrating the overlap between proteins identified by differential analysis (all significant hits) and those with VIP scores > 1. (F) Violin plots showing the expression distribution of each differentially expressed protein.

### 3.3 Correlation Between Candidate Proteins and Collateral Circulation

We compared the discriminative performance of eight candidate proteins for poor collateralization using ROC curves derived from univariable and multivariable logistic regression models. SCGB3A2 consistently yielded the highest C‐statistic, regardless of adjustment for traditional risk factors (age, BMI, gender, hypertension, smoking, eGFR, total‐to‐HDL cholesterol ratio, HbA1c, log10‐transformed hs‐CRP, and EF) (Figure 3A,B). Together with its marked differential expression and the highest VIP score, these findings identify SCGB3A2 as the leading candidate protein associated with impaired collateralization.

**FIGURE 3:**
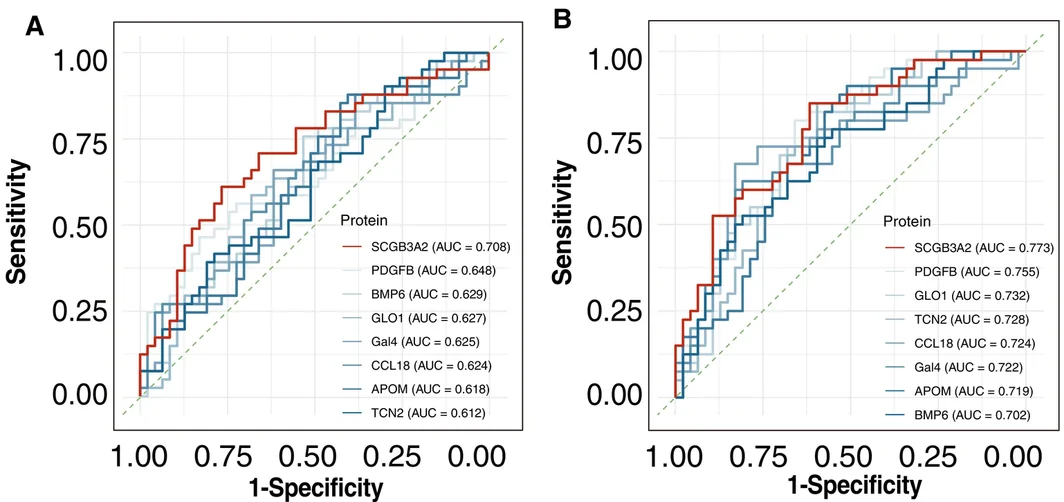
Association of candidate proteins with collateral status and SCGB3A2 distribution with rentrop score in CTO patients. ROC curves for the eight candidate proteins. (A) Unadjusted models. (B) Models adjusted for age, gender, BMI, hypertension, smoking, eGFR, total‐to‐HDL cholesterol ratio, HbA1c, log_10_‐transformed hs‐CRP, EF. BMI, body mass index; eGFR, estimated glomerular filtration rate; HbA1c, glycated hemoglobin A1c; HDL, high‐density lipoprotein; hs‐CRP, high sensitivity C‐reactive protein; SCGB3A2, secretoglobin family 3A member 2.

Further analysis of SCGB3A2 levels based on Rentrop scores revealed a negative correlation with collateralization. Patients in the highest SCGB3A2 quartile (Q4: ≥ 2.15) had a higher proportion of Rentrop score 0 (40.0%) compared to Rentrop score 3 (9.5%) (Figure S2). Conversely, patients in the lowest quartile (Q1: < 0.08) predominantly had Rentrop score 3 (47.6%) and fewer in score 0 (5.0%). This pattern indicates a negative relationship between SCGB3A2 levels and the quality of collateral circulation.

### 3.4 Validation of SCGB3A2 Protein in an Independent Patient Cohort

To validate the proteomic findings, circulating SCGB3A2 levels were quantified by ELISA in an independent validation cohort. In the T2DM cohort, patients in the PC group had higher age, greater proportions of a higher proportion of smokers and women, and elevated levels of serum creatinine, uric acid, total cholesterol, LDL cholesterol, and hs‐CRP (Table 1).

**TABLE 1:** Baseline demographic and clinical characteristics in Type 2 diabetic patients with poor and good collateralization.

Characteristics	Poor collateralization (*n* = 161)	Good collateralization (*n* = 264)	*p*
Male, *n* (%)	123 (76.4%)	231 (87.5%)	0.003
Age, y	65.96 ± 10.58	63.91 ± 9.91	0.044
Body mass index, kg/m^2^	25.13 ± 3.26	25.06 ± 2.97	0.834
Hypertension, *n* (%)	114 (70.8%)	203 (76.9%)	0.162
Smoking, *n* (%)	67 (41.6%)	83 (31.4%)	0.033
Serum creatinine, μmol/L	81.63 ± 19.79	77.18 ± 20.68	0.029
Uric acid, μmol/L	358.86 ± 115.51	332.71 ± 97.22	0.013
eGFR, mL/min per 1.73 m^2^	79.35 ± 15.86	87.08 ± 19.77	< 0.001
Triglyceride, mmol/L	1.48 (1.07–2.9)	1.47 (1.07–2.13)	0.809
Total cholesterol, mmol/L	4.25 ± 1.37	3.88 ± 1.19	0.004
HDL cholesterol, mmol/L	1.04 ± 0.27	1.04 ± 0.29	0.985
LDL cholesterol, mmol/L	2.49 ± 1.08	2.22 ± 1.01	0.010
Lipoprotein (a), g/L	0.18 (0.08–0.54)	0.17 (0.07–0.40)	0.211
Fasting blood glucose, mmol/L	7.79 ± 2.83	7.56 ± 2.56	0.404
HbA1c, %	7.31 ± 1.61	7.11 ± 1.37	0.169
hs‐CRP, mg/L	2.70 (1.00–10.57)	2.00 (0.70–5.73)	0.005
LVEF, %	56.84 ± 11.79	60.08 ± 9.32	0.002
Severity of CAD, *n* (%)			
1‐vessel	16 (9.9%)	17 (6.4%)	0.188
2‐vessel	28 (17.4%)	49 (18.6%)	0.770
3‐vessel	117 (72.7%)	198 (75.0%)	0.585
Medication, *n* (%)			
ACEI/ARB/ARNI	105 (65.2%)	180 (68.2%)	0.528
Beta‐blockers	119 (73.9%)	187 (70.8%)	0.493
Nitrates	56 (34.8%)	100 (37.9%)	0.521
Calcium channel blockers	43 (26.7%)	81 (30.7%)	0.382
Diuretic	43 (26.7%)	58 (22.0%)	0.261
Statins	153 (95.0%)	252 (95.5%)	0.841
SCGB3A2, ng/mL	19.09 (8.92–33.37)	7.503 (6.48–15.91)	< 0.001

*Note:* Values are given as mean ± SD, median (25th–75th percentile) or *n* (%). Differences between two groups were assessed using the Student's *t*‐test for normally distributed continuous variables, the Mann–Whitney *U* test for nonnormally distributed continuous variables, and the *χ* ^2^ test for categorical variables.

Abbreviations: ACEI, angiotensin‐converting enzyme inhibitor; ARB, angiotensin receptor blocker; ARNI, angiotensin receptor‐neprilysin inhibitor; eGFR, estimated glomerular filtration rate; HbA1c, glycated hemoglobin A1c; HDL, high‐density lipoprotein; hs‐CRP, high sensitivity C‐reactive protein; LDL, low‐density lipoprotein; SCGB3A2, secretoglobin family 3A member 2.

Serum SCGB3A2 levels were significantly higher in the PC group than in the GC group (median [IQR]: 19.09 [8.92–33.37] vs. 7.50 [6.48–15.91] ng/mL, *p* < 0.001) (Figure 4A) and serum SCGB3A2 levels were negatively and significantly correlated with the Rentrop score (*p* for trend < 0.001)(Figure 4B). ROC analysis showed an AUC of 0.75 (95% CI, 0.70–0.80, *p* < 0.001) for predicting poor collateralization, with an optimal cutoff of 12.56 ng/mL (sensitivity = 0.69, specificity = 0.70) (Figure 4C).

**FIGURE 4:**
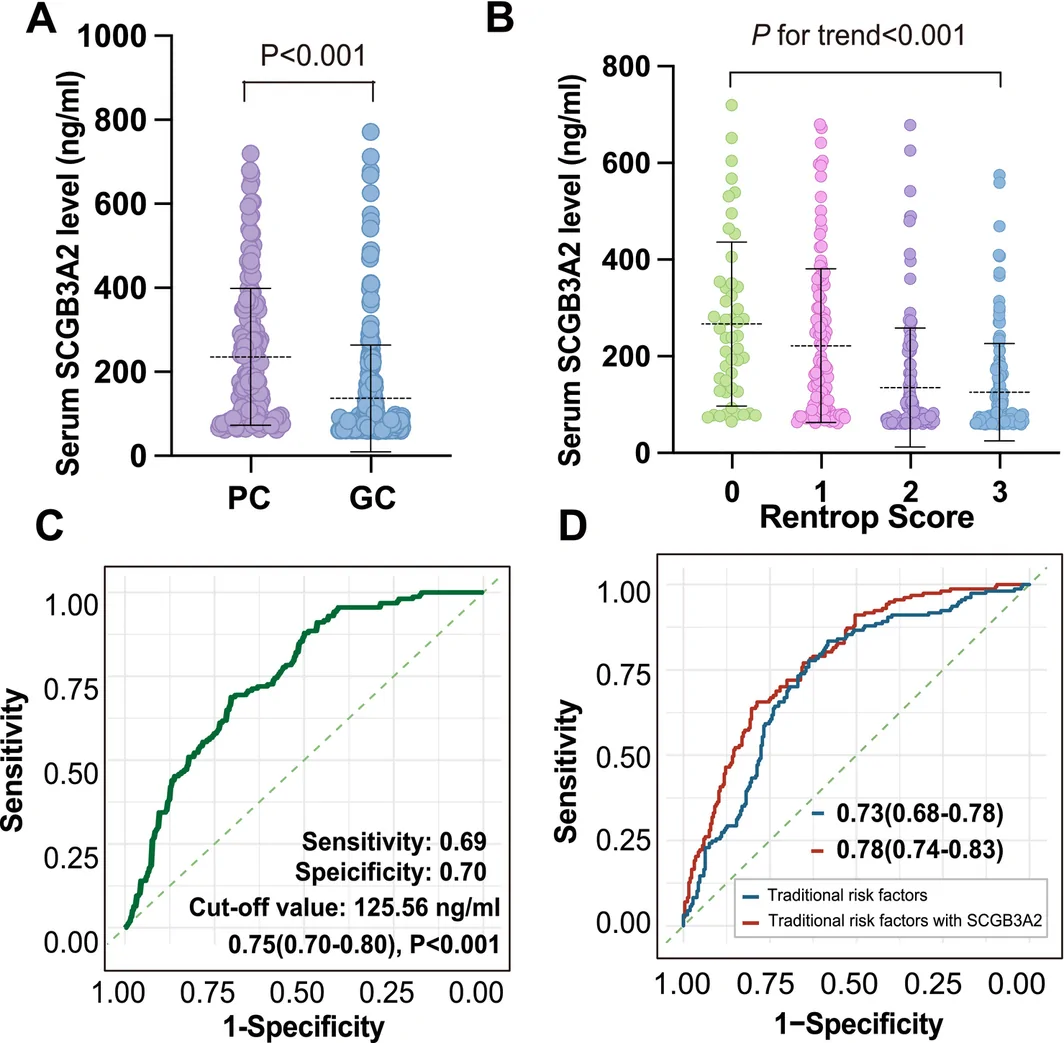
Discriminative performance of SCGB3A2 for identifying poor coronary collateralization. (A) Serum SCGB3A2 levels were significantly higher in the poor collateralization (PC) group compared to the good collateralization (GC) group. (B) A progressive increase in serum SCGB3A2 levels was observed with decreasing Rentrop collateral scores. (C) Receiver operating characteristic (ROC) curve demonstrating the discriminative performance of serum SCGB3A2 levels for identifying poor collateralization. (D) Receiver operating characteristic (ROC) curve demonstrating predicted probabilities of poor collateralization derived from a multivariable logistic regression model, adjusted for age, gender, BMI, smoking, hypertension, total‐to‐HDL cholesterol ratio, HbA1c, log10‐transformed hs‐CRP, eGFR, left ventricular ejection fraction. BMI, body mass index; eGFR, estimated glomerular filtration rate; HbA1c, glycated hemoglobin A1c; HDL, high‐density lipoprotein; hs‐CRP, high sensitivity C‐reactive protein; SCGB3A2, secretoglobin family 3A member 2.

In multivariable logistic regression analyses, serum SCGB3A2 was independently associated with poor collateralization after adjustment for established clinical risk factors, with an OR of 1.63 per 10 ng/mL increase (95% CI: 1.39–1.94; *p* < 0.001) (Table 2). Adding SCGB3A2 to the clinical model improved model fit, as reflected by an increase in Nagelkerke *R* ^2^ from 0.19 to 0.29 (Δ*R* ^2^ = 0.10), and modestly enhanced discrimination, with a 0.05 increase in the C‐statistic (both *p* < 0.001) (Figure 4D, Table 3). Furthermore, the addition of serum SCGB3A2 to the clinical model significantly improved risk reclassification and discrimination for poor collateral development, as reflected by a category‐free NRI of 0.62 (95% CI: 0.41–0.80; *p* < 0.001) and an IDI of 0.08 (95% CI: 0.05–0.11; *p* < 0.001) (Table 3). Bootstrap internal validation demonstrated limited optimism, with an optimism‐corrected C‐statistic of approximately 0.76 and a calibration slope of 0.85. Calibration analysis showed good agreement between predicted and observed probabilities, with a low maximum calibration error (*E*_max_ = 0.06) and a Brier score of 0.18 (Figure S3A). Decision curve analysis demonstrated that the model incorporating SCGB3A2 provided greater net clinical benefit across a wide range of threshold probabilities compared with the conventional clinical model (Figure S3B). Subgroup analysis in T2DM population showed consistent discriminative performance across all subgroups (*p* for interaction ≥ 0.270) (Figure 5).

**TABLE 2:** Multivariate logistic regression analyses for poor collateralization in diabetic patients.

Variables	OR (95% CI)	*p*
Model 1		
Age per 10 years	1.32 (1.05–1.67)	0.018
Gender	0.66 (0.35–1.23)	0.191
Body mass index	1.03 (0.96–1.11)	0.380
Smoking	1.85 (1.15–3.00)	0.012
Hypertension	0.72 (0.43–1.21)	0.218
Total‐to‐HDL cholesterol ratio	1.19 (1.02–1.40)	0.032
HbA1c	1.03 (0.88–1.19)	0.725
Log10‐transformed hs‐CRP	1.40 (0.99–1.97)	0.055
eGFR	0.97 (0.95–0.98)	< 0.001
Left ventricular ejection fraction	0.96 (0.94–0.98)	< 0.001
Model 2		
Age per 10 years	1.40 (1.10–1.80)	0.007
Gender	0.72 (0.37–1.40)	0.336
Body mass index	1.05 (0.98–1.14)	0.187
Smoking	1.78 (1.07–2.97)	0.027
Hypertension	0.76 (0.44–1.30)	0.314
Total‐to‐HDL cholesterol ratio	1.19 (1.00–1.41)	0.045
HbA1c	1.07 (0.91–1.25)	0.413
Log10‐transformed hs‐CRP	1.27 (0.89–1.82)	0.187
eGFR	0.96 (0.95–0.98)	< 0.001
Left ventricular ejection fraction	0.96 (0.94–0.98)	< 0.001
SCGB3A2 (per 10 ng/mL)	1.63 (1.39–1.94)	< 0.001

Abbreviations: eGFR, estimated glomerular filtration rate; HDL, high density lipoprotein; hs‐CRP, high sensitivity C‐reactive protein; SCGB3A2, secretoglobin family 3A member 2.

**TABLE 3:** Incremental predictive value of SCGB3A2 in diabetic patients.

	C‐statistics (95% CI)	ΔC‐statistics (95% CI)	DeLong *p*	Category‐free NRI (95% CI)	IDI (95% CI)
Model1	0.73 (0.68–0.78)	0.05 (0.02–0.09)	0.002	0.62 (0.43–0.81)**	0.08 (0.05–0.11)**
Model2	0.78 (0.74–0.83)				

*Note:* Model 1 excluded serum SCGB3A2 levels (per 10 ng/mL), while Model 2 included serum SCGB3A2 levels (per 10 ng/mL). Both of Model 1 and 2 included age, gender, BMI, smoking, hypertension, total‐to‐HDL cholesterol ratio, HbA1c, log10‐transformed hs‐CRP, eGFR, left ventricular ejection fraction.

Abbreviations: BMI, body mass index; eGFR, estimated glomerular filtration rate; HbA1c, glycated hemoglobin A1c; HDL, high‐density lipoprotein; hs‐CRP, high sensitivity C‐reactive protein; IDI, integrated discrimination improvement; NRI, net reclassification improvement; SCGB3A2, secretoglobin family 3A member 2.

** *p* < 0.001.

**FIGURE 5:**
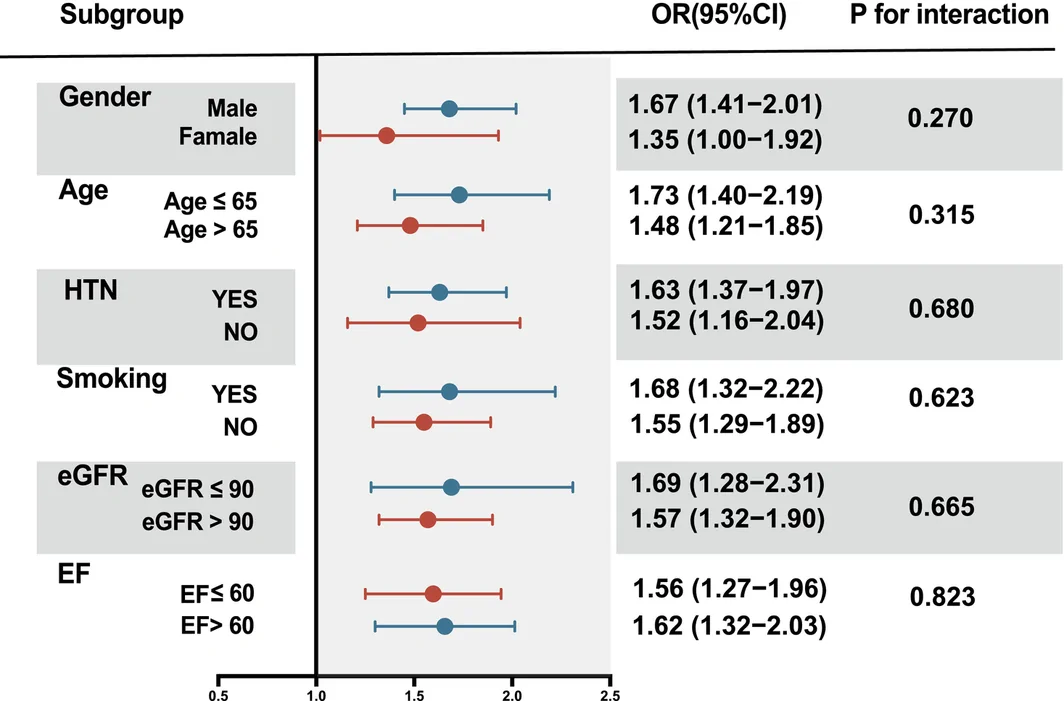
Association between SCGB3A2 and poor collateralization across clinical subgroups. Forest plot displaying adjusted odds ratios (ORs) with 95% confidence intervals for the association between serum SCGB3A2 levels (per 10 ng/mL) and poor collateralization. All models were adjusted for age, sex, and conventional cardiovascular risk factors. P for interaction ≥ 0.270 for all subgroups. SCGB3A2, secretoglobin family 3A member 2.

To explore whether the association between SCGB3A2 and collateral circulation is specific to diabetic status, we further analyzed the nondiabetic CTO cohort (*n* = 407). Consistent with the T2DM cohort, patients with poor collateralization had significantly higher serum SCGB3A2 levels than those with good collateralization (median [IQR]: 18.40 [11.65–34.20] vs. 11.89 [7.06–19.91] ng/mL; *p* < 0.001). ROC analysis demonstrated modest discriminative performance of serum SCGB3A2 for poor collateralization, with an AUC of 0.67 (95% CI: 0.62–0.73; *p* < 0.001) and an optimal cutoff value of 9.33 ng/mL. Multivariable logistic regression confirmed that SCGB3A2 remained significantly associated with poor collateralization (OR = 1.54 per 10 ng/mL, 95% CI: 1.29–1.87; *p* < 0.001). The addition of SCGB3A2 to the clinical model further improved model performance, as reflected by an increase in Nagelkerke *R* ^2^ from 0.20 to 0.27 (Δ*R* ^2^ = 0.07) and an increase in the C‐statistic of 0.04, both of which were statistically significant (both *p* < 0.001). Consistently, SCGB3A2 improved risk reclassification and discrimination, with a category‐free NRI of 0.43 (95% CI: 0.21–0.63; *p* < 0.001) and an IDI of 0.05 (95% CI: 0.03–0.08; *p* < 0.001) (Figure S4, Tables 3, S3–S5). Stratified analyses showed that the association between SCGB3A2 and impaired coronary collateralization differed according to T2DM status (*p* for interaction = 0.013; Table S6).

## 4 Discussion

In this proteomic discovery and validation study, we identified circulating SCGB3A2 as a previously unrecognized biomarker associated with impaired coronary collateralization in patients with CTO, with particular relevance to those with T2DM. SCGB3A2 was prioritized through an Olink‐based proteomic screening strategy and subsequently validated by ELISA in an independent cohort. Higher serum SCGB3A2 levels were consistently associated with poor collateralization, inversely tracked Rentrop collateral grades, and remained independently associated with impaired collateral development after adjustment for established clinical risk factors. These findings suggest that SCGB3A2 may capture aspects of collateral failure that are not fully reflected by conventional metabolic, inflammatory, or angiographic variables.

Coronary collateral development is a coordinated adaptive vascular response rather than a simple process of capillary sprouting. In the setting of CTO, progressive vessel occlusion generates hemodynamic and ischemic stimuli that may promote arteriogenesis, angiogenesis, endothelial activation, inflammatory cell recruitment, shear stress sensing, and extracellular matrix remodeling [3]. Effective collateral maturation therefore requires a balanced interaction among endothelial cells, inflammatory cells, vascular smooth muscle cells, circulating reparative cells, and the extracellular matrix. In T2DM, hyperglycemia, oxidative stress, chronic low‐grade inflammation, endothelial dysfunction, and impaired reparative cell function may collectively disrupt this adaptive response [19, 20]. These abnormalities may create a vascular environment in which the stimulus for collateral formation is present, but the biological capacity to generate mature and functional collateral vessels is insufficient.

The identification of SCGB3A2 in this setting is therefore biologically intriguing. SCGB3A2 is a secreted member of the secretoglobin family that has been mainly studied in airway epithelial biology and pulmonary injury responses [21]. Previous studies have suggested that SCGB3A2 may exert immunomodulatory, anti‐inflammatory, growth factor‐like, and anti‐fibrotic effects [22, 23, 24]. Although SCGB3A2 is not a classical vascular protein, its known biology intersects with processes relevant to collateral remodeling, including innate immune regulation, epithelial–stromal repair, extracellular matrix turnover, and tissue response to chronic injury [25]. The present findings therefore raise the possibility that impaired coronary collateralization may reflect not only local coronary factors but also a broader systemic repair‐stress phenotype.

The direction of the association deserves careful interpretation. In this study, higher circulating SCGB3A2 levels were associated with poorer collateralization. This should not be interpreted as evidence that SCGB3A2 directly impairs collateral growth. A higher circulating level of a biologically protective molecule does not necessarily indicate that the molecule is pathogenic. A comparable concept has been described for adiponectin, an adipokine with insulin‐sensitizing, anti‐inflammatory, and vasculoprotective properties, whose circulating levels may paradoxically increase in advanced cardiometabolic disease and associate with adverse outcomes [26, 27]. By analogy, elevated SCGB3A2 may represent an activated but insufficient compensatory response to chronic ischemic, inflammatory, or epithelial‐vascular stress, rather than a direct mediator of collateral failure.

This interpretation helps reconcile the apparent paradox between the reported protective properties of SCGB3A2 and its association with poor collateralization. Effective collateral growth requires a coordinated pro‐reparative response, rather than unopposed inflammation or its complete suppression [28, 29]. Excessive SCGB3A2‐related anti‐inflammatory or anti‐fibrotic signaling could theoretically dampen the inflammatory and matrix‐remodeling cues required for collateral maturation. Additionally, because SCGB3A2 can interact with SDC1, a heparan sulfate proteoglycan involved in the presentation of angiogenic factors such as VEGF and FGF, altered SCGB3A2‐SDC1 signaling may provide a potential link to impaired angiogenesis [30, 31, 32, 33]. These mechanisms remain speculative but offer testable hypotheses for future experimental studies.

The observation that similar associations were present in nondiabetic CTO patients further refines this interpretation. Although the primary focus of the present study was T2DM, the nondiabetic validation cohort showed that elevated SCGB3A2 was also associated with poor collateralization. This suggests that SCGB3A2 may reflect a broader collateral‐failure phenotype rather than a strictly diabetes‐specific biomarker. Nevertheless, T2DM may amplify this phenotype through metabolic inflammation, endothelial dysfunction, and impaired vascular repair. The numerically stronger discriminative performance in the diabetic cohort is consistent with this possibility. However, formal external validation is required to determine whether diabetes modifies the relationship between SCGB3A2 and collateral maturation.

From a clinical perspective, SCGB3A2 should not be regarded as a standalone diagnostic test at this stage. Its potential value lies in providing incremental biological information beyond traditional risk factors. In the T2DM validation cohort, serum SCGB3A2 showed moderate discrimination for poor collateralization and improved model performance when added to a clinical risk model. These findings suggest that SCGB3A2 may help identify a subgroup of CTO patients with insufficient endogenous collateral compensation, particularly among those with T2DM. However, clinical translation will require external validation, assay standardization, and prospective assessment against ischemic burden, procedural outcomes, and long‐term cardiovascular events.

In conclusion, this study identifies circulating SCGB3A2 as a novel biomarker associated with impaired coronary collateralization in CTO patients, particularly in the setting of T2DM. Rather than implying a direct pathogenic role, elevated SCGB3A2 may represent a compensatory but insufficient repair response under chronic ischemic and metabolic stress. These findings provide new insight into the biological heterogeneity of collateral failure and support further investigation of SCGB3A2 as a marker of maladaptive vascular remodeling in ischemic heart disease.

## 5 Limitations

Several limitations should be acknowledged. First, this was a single‐center observational study; therefore, although serum SCGB3A2 was independently associated with poor coronary collateralization, causal relationships cannot be inferred. Residual confounding from unmeasured clinical, metabolic, inflammatory, or angiographic factors may still exist despite multivariable adjustment.

Second, coronary collateral circulation was assessed using the Rentrop score, which is a widely used but semi‐quantitative angiographic grading system. This method may be influenced by image quality, contrast injection, hemodynamic conditions, and interobserver variability. More quantitative approaches, such as collateral flow index or advanced perfusion imaging, may provide additional validation.

Third, the present study did not directly investigate the biological mechanisms linking SCGB3A2 to impaired collateral development, nor did it evaluate whether SCGB3A2 predicts long‐term clinical outcomes such as successful revascularization, recurrent ischemia, heart failure, or major adverse cardiovascular events. Future experimental and prospective outcome studies are needed to clarify the mechanistic and prognostic significance of SCGB3A2 in patients with CTO and T2DM.

Forth, detailed information on diabetic microvascular complications was not systematically available in the discovery cohort, precluding adjustment for or stratified analyses according to microvascular disease.

What's more, although the AUC provides an overall measure of discrimination, the optimal cutoff value derived from ROC analysis may be influenced by the distribution of the outcome in the study population. Therefore, the cutoff values identified in the present study should be considered cohort‐specific estimates rather than universal clinical thresholds and should be externally validated in independent populations.

## Author Contributions

J.C. performed the experiments, collected and analyzed the data, and drafted the manuscript. S.C. assisted with data collection and data analysis. F.‐H.D. and Y.D. conceived and designed the study. F.‐H.D. supervised the research and critically revised the manuscript. F.‐H.D. and Y.D. acquired funding. Y.D. also contributed to revising the manuscript. All authors reviewed and approved the final manuscript.

## Funding

This study was supported by the National Natural Science Foundation of China (82270425, 82470433) and Science and Technology Commission of Shanghai Municipality (24ZR1446100, 25ZR1402331).

## Conflicts of Interest

The authors declare no conflicts of interest.

## Supporting information


**Figure S1:** PLS‐DA model characteristics and permutation validation.
**Figure S2:** Stacked bar of SCGB3A2 NPX within each Rentrop score group.
**Figure S3:** Calibration and decision curve analyses of the multivariable prediction model.
**Figure S4:** ROC curve demonstrating the diagnostic accuracy of serum SCGB3A2 levels (per 10‐ng/L) for identifying poor collateralization in nondiabetic patients.
**Table S1:** Baseline demographic and clinical characteristics in T2DM patients of discovery cohort with PC and GC.
**Table S2:** Significantly elevated or reduced biomarkers among the 265 measured by multiplex PEA.
**Table S3:** Baseline demographic and clinical characteristics in nondiabetic patients with PC and GC.
**Table S4:** Multivariate logistic regression analyses for poor collateralization in nondiabetic patients.
**Table S5:** Incremental predictive value of SCGB3A2 in nondiabetic patients.
**Table S6:** Interaction analysis between SCGB3A2 and T2DM status for impaired coronary collateralization.

## Data Availability

The data that support the findings of this study are available from the corresponding author upon reasonable request.
