# Supplementary material for: Circulating SCGB3A2 Is Associated With Impaired Coronary Collateralization in Patients With Chronic Total Occlusion and Type 2 Diabetes: A Proteomic Discovery and Validation Study
Source: J Diabetes. 2026 Oct 15;18(10):e70281. doi: 10.1111/1753-0407.70281 (PMC13671098; doi:10.1111/1753-0407.70281)
Supplement: Supplementary file 1 [file JDB-18-e70281-s001.docx]

Supplementary material

**Figure S1. PLS-DA model characteristics and permutation validation.**

(A) Loading plot showing the contribution of individual proteins to the first two components in the PLS-DA model.

(B) Distribution of balanced error rates (BERs) generated from 1000 permuted PLS-DA models. PLS-DA, partial least squares discriminant analysis.

**Figure S2. Stacked bar of SCGB3A2 NPX within each Rentrop score group.**

Data are presented as percentages of the total number of patients per Rentrop score.

NPX, normalized protein expression; SCGB3A2, secretoglobin family 3A member 2.

**Figure S3. Calibration and decision curve analyses of the multivariable prediction model.**

(A)Bootstrap calibration curve of model 2 for predicting poor coronary collateralization. Calibration analysis was performed using 1000 bootstrap resamples.

(B) Decision curve analysis comparing the multivariable models with and without SCGB3A2.

Model 1 excluded serum SCGB3A2 levels (per 10-ng/mL), while model 2 included serum SCGB3A2 levels (per 10-ng/mL). Both of model 1 and 2 included age, gender, BMI, smoking, hypertension, total-to-HDL cholesterol ratio, HbA1c, log10-transformed hs-CRP, eGFR, left ventricular ejection fraction.

BMI, body mass index; eGFR, estimated glomerular filtration rate; HbA1c, glycated hemoglobin A1c; HDL, high-density lipoprotein; hs-CRP, high sensitivity C-reactive protein; IDI, integrated discrimination improvement; NRI, net reclassification improvement; SCGB3A2, secretoglobin family 3A member 2.

**Figure S4. ROC curve demonstrating the diagnostic accuracy of serum SCGB3A2 levels (per 10-ng/L) for identifying poor collateralization in non-diabetic patients.**

(A) Predicted probabilities of poor collateralization derived from a multivariable logistic regression model (B), adjusted for age, gender, BMI, smoking, hypertension, total-to-HDL cholesterol ratio, HbA1c, log10-transformed hs-CRP, eGFR, left ventricular ejection fraction.

BMI, body mass index; eGFR, estimated glomerular filtration rate; HbA1c, glycated hemoglobin A1c; HDL, high-density lipoprotein; hs-CRP, high sensitivity C-reactive protein; ROC,Receiver operating characteristic; SCGB3A2, secretoglobin family 3A member 2.

| **Table S1. Baseline demographic and clinical characteristics in T2DM patients of discovery cohort with PC and GC** | | |
| --- | --- | --- |
| **Characteristic** | **PC (n=41)** | **GC (n=47)** |
| Male, n (%) | 34 (82.9%) | 37 (78.7%) |
| Age, y | 67.27±10.07 | 64.28±10.50 |
| Body mass index, kg/m^2^ | 25.52±2.91 | 25.04±2.61 |
| Hypertension, n (%) | 28 (68.3%) | 34 (72.3%) |
| Smoking, n (%) | 17 (41.4%) | 15(31.9%) |
| Serum creatinine, μmol/L | 93.41±50.93 | 83.36±21.98 |
| Uric acid, umol/L | 359.59±121.91 | 354.96±88.64 |
| eGFR, mL/min per 1.73m^2^ | 78.29±22.46 | 82.20±17.50 |
| Triglyceride, mmol/L | 1.41 (1.12-2.12) | 1.30 (1.06-2.14) |
| Total cholesterol, mmol/L | 4.28±1.29 | 3.69±0.98 |
| HDL-cholesterol, mmol/L | 1.07±0.25 | 0.99±0.23 |
| LDL-cholesterol, mmol/L | 2.59±1.07 | 2.09±0.86 |
| Lipoprotein (a), g/L | 0.22 (0.09-0.41) | 0.11 (0.05-0.35) |
| Fasting blood glucose, mmol/L | 7.68±3.22 | 6.77±2.69 |
| HbA1c, % | 7.03±1.14 | 6.98±1.25 |
| hs-CRP, mg/L | 1.94 (0.55-5.27) | 1.00 (0.43-6.78) |
| Left ventricular ejection fraction, % | 55.43±12.40 | 57.49±12.76 |
| Severity of CAD, n (%) | | |
| 1-vessel | 5(12.2%) | 3(6.4%) |
| 2-vessels | 7(17.1%) | 6(12.8%) |
| 3-vessels | 29(70.7%) | 38(80.9%) |

Values are given as mean±SD, median (25th-75th percentile) or n (%).

eGFR, estimated glomerular filtration rate; HbA1c, glycated hemoglobin A1c; HDL, high-density lipoprotein; LDL, low-density lipoprotein; SCGB3A2, secretoglobin family 3A member 2.

| **Table S2. Significantly elevated or reduced biomarkers among the 265 measured by multiplex PEA** | | | |
| --- | --- | --- | --- |
| Biomarker | Log2-fold change | p value | FDR-adjusted P values |
| SCGB3A2 | 0.54 | 1.44×10^-3^ | 0.382 |
| PDGFB | -0.48 | 8.40×10^-3^ | 0.920 |
| TCN2 | 0.13 | 2.66×10^-2^ | 0.920 |
| CCL18 | 0.32 | 3.54×10^-2^ | 0.920 |
| LGALS4 | 0.25 | 3.65×10^-2^ | 0.920 |
| APOM | 0.17 | 4.87×10^-2^ | 0.920 |
| BMP6 | 0.20 | 4.97×10^-2^ | 0.920 |
| GLO1 | -0.56 | 4.98×10^-2^ | 0.920 |

All Biomarker names are according to HUGO gene nomenclature committee (HGNC) nomenclature.

| **Table S3. Baseline demographic and clinical characteristics in non-diabetic patients with PC and GC** | | | |
| --- | --- | --- | --- |
| **Characteristic** | **PC (n=128)** | **GC (n=279)** | **p** |
| Male, n (%) | 105 (82.0%) | 242 (86.7%) | 0.214 |
| Age, y | 65.77±11.94 | 62.58±11.76 | 0.012 |
| Body mass index, kg/m^2^ | 25.42±3.79 | 25.20±3.21 | 0.551 |
| Hypertension, n (%) | 97 (75.8%) | 199 (71.3%) | 0.349 |
| Smoking, n (%) | 56 (43.8%) | 86 (30.8%) | 0.011 |
| Serum creatinine, μmol/L | 79.74±17.51 | 78.03±18.46 | 0.380 |
| Uric acid, umol/L | 361.98±106.65 | 356.97±97.26 | 0.641 |
| eGFR, mL/min per 1.73m^2^ | 81.98±14.50 | 87.10±19.21 | 0.008 |
| Triglyceride, mmol/L | 1.38 (1.10-1.88) | 1.29 (0.97-1.77) | 0.103 |
| Total cholesterol, mmol/L | 4.40±1.52 | 4.01±1.09 | 0.003 |
| HDL-cholesterol, mmol/L | 1.03±0.24 | 1.05±0.23 | 0.668 |
| LDL-cholesterol, mmol/L | 2.60±1.19 | 2.40±1.01 | 0.075 |
| Lipoprotein (a), g/L | 0.21 (0.09-0.49) | 0.19 (0.09-0.51) | 0.941 |
| Fast blood glucose, mmol/L | 5.17±0.57 | 5.16±0.52 | 0.904 |
| HbA1c, % | 5.76±0.48 | 5.71±0.46 | 0.328 |
| hs-CRP, mg/L | 2.62 (0.96-10.21) | 1.98 (0.73-4.00) | 0.002 |
| Severity of CAD, n (%) | | | |
| 1vessel | 20 (15.6%) | 27 (9.7%) | 0.084 |
| 2vessels | 21 (16.4%) | 59 (21.1%) | 0.257 |
| 3vessels | 87 (68.0%) | 193 (69.2%) | 0.825 |
| Medication, n (%) | | | |
| ACEI/ARB/ARNI | 85 (62.3%) | 175 (62.9%) | 0.473 |
| Beta-blockers | 90 (69.2%) | 186 (67.6%) | 0.465 |
| Nitrates | 48 (32.7%) | 93 (35.3%) | 0.412 |
| Calcium channel blockers | 28 (20.8%) | 85 (31.5%) | 0.072 |
| Diuretic | 30 (18.2%) | 50 (22.4%) | 0.194 |
| Statins | 124(96.9%) | 272(97.5%) | 0.722 |
| SCGB3A2, ng/mL | 18.40 (11.65-34.20) | 11.89 (7.06-19.91) | <0.001 |

Values are given as mean±SD, median (25th-75th percentile) or n (%). Differences between two groups were assessed using the Student’s t-test for normally distributed continuous variables, the Mann-Whitney U test for non-normally distributed continuous variables, and the χ² test for categorical variables.

ACEI, angiotensin-converting enzyme inhibitor; ARB, angiotensin receptor blocker; ARNI, angiotensin receptor-neprilysin inhibitor; eGFR, estimated glomerular filtration rate; HbA1c, glycated hemoglobin A1c; HDL, high-density lipoprotein; LDL, low-density lipoprotein; SCGB3A2, secretoglobin family 3A member 2 .

| **Table S4. Multivariate logistic regression analyses for poor collateralization in non-diabetic patients** | | |
| --- | --- | --- |
| Variables | OR (95% CI) | p value |
| Model 1 | | |
| Age per 10 years | 1.33 (1.06-1.68) | 0.014 |
| Gender | 0.62 (0.29-1.32) | 0.210 |
| Body mass index | 1.05 (0.98-1.13) | 0.188 |
| Smoking | 2.08 (1.25-3.50) | 0.005 |
| Hypertension | 1.45 (0.83-2.58) | 0.197 |
| Total-to-HDL cholesterol ratio | 1.24 (1.06-1.46) | 0.008 |
| HbA1c | 1.03 (0.61-1.70) | 0.915 |
| Log10-transformed hs-CRP | 1.45 (1.00-2.12) | 0.050 |
| eGFR | 0.97 (0.96-0.99) | 0.005 |
| Left ventricular ejection fraction | 0.95 (0.93-0.98) | <0.001 |
| Model 2 | | |
| Age per 10 years | 1.30 (1.03-1.65) | 0.030 |
| Gender | 0.58 (0.27-1.26) | 0.163 |
| Body mass index | 1.07 (0.99-1.15) | 0.073 |
| Smoking | 2.39 (1.39-4.15) | 0.002 |
| Hypertension | 1.44 (0.81-2.60) | 0.221 |
| Total-to-HDL cholesterol ratio | 1.21 (1.03-1.42) | 0.021 |
| HbA1c | 1.14 (0.66-1.92) | 0.631 |
| Log10-transformed hs-CRP | 1.31 (0.89-1.95) | 0.175 |
| eGFR | 0.98 (0.96-0.99) | 0.009 |
| Left ventricular ejection fraction | 0.95 (0.93-0.98) | <0.001 |
| SCGB3A2 (per 10-ng/mL) | 1.54 (1.29-1.87) | <0.001 |

eGFR, estimated glomerular filtration rate; hs-CRP, high sensitivity C-reactive protein; SCGB3A2, secretoglobin family 3A member 2; TC/HDL, total cholesterol to high-density lipoprotein ratio.

| **Table S5. Incremental predictive value of SCGB3A2 in non-diabetic patients** | | | | | |
| --- | --- | --- | --- | --- | --- |
|  | **C-statistics (95% CI)** | **ΔC-statistics (95% CI)** | **DeLong P** | **Category-free NRI (95% CI)** | **IDI (95% CI)** |
| Model1 | 0.73 (0.67-0.78) | 0.04 (0.01-0.07) | 0.007 | 0.43 (0.21-0.63)^**^ | 0.05 (0.03-0.08)^**^ |
| Model2 | 0.77 (0.72-0.82) |  |  |  |  |

Significance: ^**^p<0.001.

Model 1 excluded serum SCGB3A2 levels (per 10-ng/mL), while Model 2 included serum SCGB3A2 levels (per 10-ng/mL). Both of Model 1 and 2 included age, gender, BMI, smoking, hypertension, total-to-HDL cholesterol ratio, HbA1c, log10-transformed hs-CRP, eGFR, left ventricular ejection fraction. BMI, body mass index; eGFR, estimated glomerular filtration rate; HbA1c, glycated hemoglobin A1c; HDL, high-density lipoprotein; hs-CRP, high sensitivity C-reactive protein; IDI, integrated discrimination improvement; NRI, net reclassification improvement; SCGB3A2, secretoglobin family 3A member 2.

| **Table S6. Interaction analysis between SCGB3A2 and T2DM status for impaired coronary collateralization** | | | |
| --- | --- | --- | --- |
| T2DM | OR | P.value | P for interaction |
| Yes (n=425) | 2.23 (1.81–2.76) | <0.001 | 0.013 |
| No (n=407) | 1.59 (1.35–1.88) | <0.001 |  |

Odds ratios (ORs) with 95% confidence intervals for the association between serum SCGB3A2 levels (per 10-ng/mL) and poor collateralization.
